# Imagine: An Interview with Svante Pääbo

**DOI:** 10.1371/journal.pgen.1000035

**Published:** 2008-03-28

**Authors:** Jane Gitschier

**Affiliations:** Departments of Medicine and Pediatrics, Institute for Human Genetics, University of California San Francisco, San Francisco, California, United States of America

Svante Pääbo works on the edge of what's possible. He ignites our imagination, unlocking tightly held secrets in ancient remains. By patiently and meticulously working out techniques to extract genetic information from skin, teeth, bones, and excrement, Pääbo has become the leader of the ancient DNA pack. Sloths, cave bears, moas, wooly mammoths, extinct bees, and Neanderthals—all have succumbed to his scrutiny.

**Image 1:**
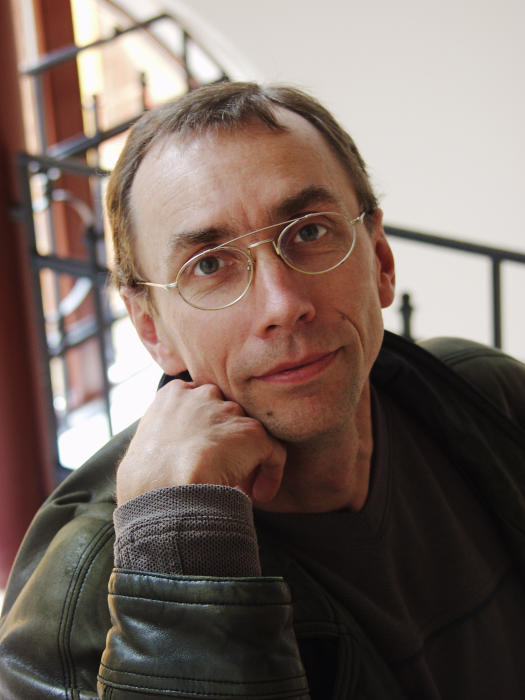
Svante Pääbo

Pääbo (see Image 1) broke ground in 1985, working surreptitiously at night in the lab where he conducted his unrelated PhD research, to extract, clone, and sequence DNA from an Egyptian mummy. From there, he joined the late Allan Wilson as a post-doctoral fellow in Berkeley, where together they rejuvenated sequences from extinct species. Returning to Europe, he landed a full professor position in Munich. He is now Director of Evolutionary Genetics at the Max Planck Institute for Evolutionary Anthropology in Leipzig.

A sterile hotel lobby wasn't the venue I had hoped for in interviewing Pääbo. I would have preferred a natural history museum, or, even better, an archaeological dig to stimulate the interview juices. But, when I realized he was attending the American Society of Human Genetics meeting in San Diego last fall, I grabbed the opportunity. Though jet-lagged, he gamely agreed to a 10 p.m. interview, following the Presidential symposium and only seven hours prior to his planned surfing excursion in La Jolla.

**Jane Gitschier: What happened in your youth to make you so interested in Egypt?**

**Svante Pääbo:** Sometime in my late boyhood, I got very interested in archeology. I went around after big storms in Sweden to spots in which trees had fallen over. You can look at the roots for things—stone age pottery and things like that. Even in the suburbs of Stockholm, where I grew up, there was still a forest around. And you could run around and have fun. It certainly was common for kids to play “stone age” behind the school in the forest.

**JG: Was there something that triggered your particular interest in archeology?**

**SP:** Not really, but I think it was the realization that you *could* actually go out yourself and find these things!

**JG: And did you find stuff?**

**SP:** Yes, they are still at my mother's place, in a glass cabinet—thousands of pot shards that I collected. You can sometimes passel them together and can get part of a pot that was used 3,000 years ago. Quite fascinating.

Also, my mother had taken me to Egypt because I was interested in Egyptology. I think I was 14. That made me fascinated, as so many young kids are, with Egypt and mummies and pyramids. It was mainly the trips I took to Egypt—three times with my mom.

**JG: Wow, was your mother into Egypt, too?**

**SP:** It was partially through my fascination, but I think she still goes to lectures on Egyptology in Stockholm.

**JG: Were your parents scientists?**

**SP:** Yes. I grew up with my mother. My mom and dad were not married. My mom was a chemist and worked in industry. My dad had another family, but he was a biochemist and studied prostaglandins.

**JG: And then you worked in biochemistry?**

**SP:** I first started studying Egyptology and things like that at the University [Uppsala] and got somehow disappointed. It was not as romantic as I thought it would be. And after a year and a half or so, I didn't know what to do, because this wasn't really “it”. So I started studying medicine because I figured I would get a profession. And it was also a way into basic research.

**JG: I read your paper from 1985 about sequencing the mummy remains. What was the genesis of that?**

**SP:** I knew there were hundreds and thousands of mummies around in museums and that they found hundreds of new ones every year, and molecular cloning in bacteria was a rather new thing at the time, so I found in the literature that no one had tried to extract DNA from Egyptian mummies, or any old remains actually. So I started to do that as a hobby in late evenings and weekends, secretly from my thesis advisor.

**JG: As a lowly graduate student, where do you find a piece of mummy to start this investigation?**

**SP:** I had studied Egyptology, so the professor of Egyptology knew me quite well. He helped me to sample a mummy in the museum in Uppsala. He also had very good connections with a very large museum in Berlin, which was East Berlin at the time. Germany has a long, long tradition in Egyptology, going back to the 19^th^ century. After the British Museum and the Museum in Paris, the Berlin Museum has the biggest collection outside Egypt.

**JG: So you went with your professor to the museum in East Berlin…**

**SP:** He had convinced them of our idea in advance. We sampled, I think, 36 different mummies. Small samples, of course.

**JG: Had people ever looked at mummy tissue before, at things like proteins?**

**SP:** There had been some work on histology of mummies, and there had been some work on trying immunoreactivity of proteins extracted from it, with very mixed results. I don't think there were any convincing results from Egyptian mummies.

**JG: In what kind of state are the mummies? Are you wearing gloves or masks? What are you doing?**

**SP:** We only worked with mummies that were already unwrapped and with things that were broken, so we were not destroying anything to get to the tissues. With a scalpel we removed a little piece. It was the first time this was done, so we had no big qualms about contamination. I had no idea this could be such a big issue.

**JG: What did you do with these 36 scalpeled samples?**

**SP:** We screened them with histology. We looked at both with traditional stainings—hematoxylin-and-eosin staining and staining with ethidium bromide—and under UV light to see if one could see any fluorescence from DNA. In the skin of a particular mummy, you could see that the cell nuclei lit up. So, there was DNA there and at the place you would expect it to be.

**JG: Was your interest in this simply the challenge of getting DNA sequence out of it or was there a bigger idea?**

**SP:** It was clearly the idea that if you could study the DNA of ancient Egyptians, you could elucidate aspects of Egyptian history that you couldn't by traditional sources of archeology and the written records.

**JG: Do you mean the relationships between people?**

**SP:** Population history. Say, when Alexander the Great conquered Egypt, did that mean there were lots of people from Greece who actually came there and settled there? When the Assyrians came there, did that have an influence? Or was the population continuous? Political things that influenced the population.

Since then it has become clear that it is almost impossible to work with human remains because of contamination. It is very hard to exclude that the DNA you look at is not contaminated with modern humans.

**JG: Then, how do we know that this sequence in the 1985 paper is in fact the sequence of a real Egyptian?**

**SP:** In hindsight, we don't know that. In 1985, I had no idea how hard this is [to retrieve uncontaminated ancient DNA sequences] and thus did not do the controls we now know are necessary. We've even published at a later point on this.

**JG: But there have been no data to refute the sequence of this mummy.**

**SP:** But nothing to prove it either! It could well have been contamination, and if that was the last that had ever been written on ancient DNA, that would have been a sad state of affairs and the end of the field.

**JG: Have people gone on to look at more mummy DNA since then?**

**SP:** Egyptian mummies are actually quite badly preserved; also animal mummies. This probably has to do with climate. It seems the cooler it is, the better preserved things are. We looked at a few Neanderthal remains from Israel and Palestine and they have so far not yielded any DNA.

**JG: What is it like to travel all over the world to try to get specimens?**

**SP:** To sample these things takes building confidence—in museum curators and archeologists and paleontologists—that we *can* actually get information from them. And, of course, it is a balance for a curator between a destructive sampling for scientific progress against responsibility for future generations to preserve these things. With justification, you can sometimes say that if you can just wait 30 years, methods will be so much better.

What you actually do is a several stage process, where you first take very small samples, of say 10 mg, and just see if there are amino acids preserved—the amino acid profile of collagen. If there is no collagen preservation, it turns out there is hardly ever DNA. We can already exclude a lot of remains that way.

And then we take samples of 100–200 mg, extract DNA, and see if we can find Neanderthal DNA. And then for the genome project where we need larger samples, we use bones that have very little morphological information. So in the Museum in Zagreb, which houses the Vindija remains, we screen bones of which it cannot be said from the morphology if they are human or animal. By doing extraction from 100 mg, you can determine the species from the mitochondrial DNA. So the paleontologists gain something—they learn what species the different bones come from, and so it is easy to justify taking half a gram from them if they turn out to be Neanderthal bones.

**JG: So now, back to the mummies. That was not your thesis.**

**SP:** No, but then I had to tell my thesis advisor that I had done this! He was happy that it had been successful. I don't think he would have been so happy if I had presented it before it happened.

**JG: Then you went to Allan Wilson's lab. What did you work on there?**

**SP:** Really developing the technology for ancient DNA. PCR had just come around, and I had tried to do PCR back in Europe with water baths. It was really when Taq polymerase came and the thermocycler, and Allan's lab was, I think, the first academic lab to have one.

**JG: And what organism were you working on?**

**SP:** We started again on Egyptian mummies but rather soon switched to animals of different sorts. We worked on moas from New Zealand, which are extinct flightless birds, ground sloths, and the marsupial wolf from Australia.

**JG: Why did you switch? Better preserved or no contamination issues?**

**SP:** Contamination with human DNA became apparent rather soon when I started using PCR, when you could repeat experiments and do lots of negative controls.

**JG: Was your passion, though, in these extinct animals? Or was your intent to go back to human lineages?**

**SP:** It took on its own life. It became very fascinating to develop the technology and overcome the problems with contamination, problems with errors in the sequences, things like that.

**JG: How did your move to Germany come about?**

**SP:** Pretty much by chance. I had a girlfriend—I've had both boyfriends and girlfriends in my life—but at some point I had a girlfriend who was from Munich. The professor of genetics there asked me to give a seminar at some point, and then he said they had this professorship coming up in a year and I ought to apply, which I did, and by the time it had all worked out … I had no girlfriend there anymore!

But, it was clearly a very, very good offer. The biggest break I got in my life. I became a full professor there after being a post-doc, directly, without being an assistant professor. The opposite to your prejudice about how European science works, in that case. There was a constellation of people there who were not risk-averse.

**JG: How did the Institute in Leipzig come to be?**

**SP:** After German reunification, there was the political will and the money to start institutes in East Germany at the same density, according to population, as there were in West Germany. This was the chance to start a number of new Max Planck Institutes. And there was a very conscious idea to ask—in what areas of science is Germany particularly weak? And of course, anthropology is such.

**JG: They were weak in anthropology?**

**SP:** Absolutely. Due to what happened during Nazi times. There had been an Institute of Anthropology where Mengele was an assistant. And so no one had really wanted to touch anthropology since that time.

So there was a lot discussion if one would dare to do it or if it was too politically sensitive. And then, once the decision was made to actually do something in the direction of human evolution, it was in fact a big advantage that there were no big traditions. Because you could say—how would we *now* start an institute in evolutionary anthropology not burdened by any traditions? And the idea sort of grew among people who discussed this. If we were to do this, we would ask the question of what makes humans unique in a comparative way across different disciplines—humanities or sciences—but it should all be empirical, not just a question of philosophy.

It ended up being an institute with five departments: Paleontology; Primatology, with research sites in Africa, studying chimps and gorillas in their natural habitats and their range of behaviors; Comparative Psychology, which has a primate facility in Leipzig, the only research facility in the world with all the great apes, and it's part of the zoo. Visitors can actually observe the experiments. They do experiments in cognitive development in human children and ape children for the first 12 months of life—the very same experiments. When do you see the things that set humans apart and what are these things? And there is Comparative Linguistics—what is common to all human languages? And then Genetics.

**JG: What have you got your eye on, other than Neanderthals?**

**SP:** We are very interested in comparative genomics of the apes in general. We are sequencing the bonobo—the last ape that has not been sequenced—with the 454 technology. The amazing thing is with these high-throughput technologies, a lab can now take on projects that a genome center did just a few years ago.

For the Neanderthal, we have to do so much sequencing that we do it with the 454 company in a collaboration. We test the libraries we make in the clean room, and when we have a good library, it goes to Bradford [Connecticut] for the production sequencing.

**JG: Do people come to you with crazy ideas that intrigue you?**

**SP:** Yes. Our lab pretty much functions on the ideas that are born in the group, and our lab is a little unusual in that we spend a lot of time discussing every project every week, in a group setting. All the groups that have to do with ancient DNA—all the people sit together once a week and discuss their work—particularly things that don't work. Gene expression is another day, or genomics, and it is in these sorts of eternal discussions that ideas come. It is quite rare that anyone sitting alone thinking in their room comes up with any big ideas. It's really by throwing lots of ideas around. If you have a hundred ideas on the table, then one of them turns out to be really cool.

**JG: What other mysteries would you like to address?**

**SP:** What one dreams about is defining the genetic changes that we all share today but that made modern humans so special. That made us colonize the whole place, every little speck of land on the planet, which, after all, archaic humans had not done. They had been around for two million years, but they never crossed the water where they couldn't see land on the other side. Modern humans have been around for a hundred thousand years and we've colonized Easter Island, right?

**JG: Not to mention we went to the moon.**

**SP:** Exactly! We're crazy. Nothing really stops us. So there is something really special there in how we behave—to somehow understand that!

Something we also talk a lot about in the group these days is how genetic diversity is structured in humans. I think we are still far too much in the pattern of looking at diversity of different groups and the boundaries between them because of how we have sampled and how we have looked at things. I think, in a way, it is sad that people interested in population history have gone out and sampled according to preconceived ideas of what groups are there, be those linguistic groups or racial groups, and of course if you sample like that you come up with some differences between groups, and say yes, they are there. Rather than going out and just sampling without regard for anything other than geography.

**JG: So you mean, just getting a map and sampling a person at every grid point.**

**SP:** Yes, and the logistics of doing that over a whole continent are almost impossible.

But coming back to Egypt again, what I would really like to do is have a boat and sail along the Nile from the Mediterranean, where people are really “European-like” to the source of the Nile in Lake Victoria, where people are really “African-like”, and sample every 50 kilometers along this corridor through the Sahara and just see how this transition occurs. Are there sharp borders or is there a gradient? And that would be a powerful thing to look at, and it would be feasible.

**JG: Well, if Craig Venter can sail around the world collecting microbial samples, you should be able to get a boat for a trip up the Nile.**

**SP:** I'm not as independently wealthy as he is!

**JG: Now, in our final moments, I want to ask what has been your favorite project.**

**SP:** I tend to think the current project is the favorite project. Every project has this manic thing about enormous expectations in it, that often are not borne out to the extent that you imagine, but it's what drives it. And then you come down to the reality of things.

But clearly now, I would say, being able to see the Neanderthal genome is something that just a couple of years ago I wouldn't think would be possible in my lifetime. And now, it is.

**JG: What do you think it is about Neanderthals that excites people?**

**SP:** Quite recently, only some 2,000 generations or so ago, there were some other humans with us who were similar, but clearly distinct from us. It gives us some perspective.

Sometimes I like to make the thought experiment—that they made it another 2,000 generations and were here! What consequences would that have? Would racism against Neanderthals have been even worse than the sort of racism we experience today, because they truly were a bit different, or is it that if we had had something like that that was another human form, then perhaps we wouldn't have been able to distance ourselves so much from the great apes as we do now —not making this enormous distinction we do now between what we call humans and all other organisms which we call animals.

It could have gone either way—we can never know, but these are things it is interesting to think about because it puts these issues in our society in perspective. Perhaps somewhere there is the fascination.

